# PReS-FINAL-2072: How does the management of enthesitis related arthritis differ from other sub-types of juvenile idiopathic arthritis? A retrospective study of jia at an adolescent rheumatology centre

**DOI:** 10.1186/1546-0096-11-S2-P84

**Published:** 2013-12-05

**Authors:** M Choudhury, D Sen, J Ioannou

**Affiliations:** 1 Arthritis Research UK Centre for Adolescent Rheumatology, London, UK; 2 Medical School, University College London, London, UK; 3 Paediatric, Adolescent & Adult Rheumatology, London, UK; 4 Adolescent and Adult Rheumatology, Great Ormond St Hospital & University College London Hospitals NHS Foundation Trusts, London, UK

## Introduction

Enthesitis Related Arthritis (ERA) is a sub-type of JIA with emerging phenotypic differences. Clinical onset is often insidious and there is evidence to suggest long-term remission is less common. There is limited data comparing the treatment of ERA to other sub-types for the adolescent age spectrum.

## Objectives

To determine if DMARD and biologic use differed in patients with ERA compared to other sub-types of JIA during adolescence.

## Methods

A detailed retrospective review of medical notes and referral documentation for all JIA patients (N = 159) attending an Adolescent Rheumatology Centre over a three month period was made.

## Results

The median age overall was 17.1(13-21) years. 28.9% (46) of individuals had ERA, of which 69.6% (39) were male. Mean duration of disease since diagnosis was 7.3 years (SD=+/-4.9). Of the other JIA subtypes; 21.4% (34), 17.6% (28) and 11.9% (19) had Extended Oligoarticular Arthritis (EOA), Polyarticular Rheumatoid Factor Positive (JIA RF+) and Polyarticular Rheumatoid Factor Negative (JIA RF-) JIA, respectively. There was no significant difference in mean time since diagnosis between ERA and the other JIA subgroups.

The time between diagnosis and commencing a DMARD, primarily Methotrexate, was significantly longer in the ERA group (31.6 vs. 22.5 months, p = 0.008). Mean duration on Methotrexate was significantly shorter in ERA compared to EOA and JIA RF+ subgroups (31 vs. 47 and 49 months respectively, p = 0.03). 80.4% of ERA patients had been started on Methotrexate since diagnosis, with 63% continuing it at the time of the study. 57.14% of those that had stopped were discontinued due to poor treatment response. This was substantially higher than EOA and JIA RF- (36.6% and 25% respectively). 17% of ERA patients had had Sulfasalazine treatment in the past compared to 5.7% and 5.8% of Polyarticular RF (polya) and EOA, respectively. The duration of Sulfasalazine treatment was significantly longer when compared with polya and EOA (36 months vs. 23 and 5 months, respectively, p = 0.034).

At the time of study, a greater proportion of ERA patients were on biological treatment when compared with polya and EOA (38.9% vs. 30.7% and 17.6%, respectively, p < 0.001). 10.8% of ERA were using Infliximab compared with 0% of EOA and 1.9% of polya (p < 0.001). Adalimumab use was more prevalent amongst ERA compared with EOA and polya (13% vs. 0% and 3.8%, respectively, (p = 0.008). The time from starting a DMARD to starting a biologic was significantly shorter in ERA compared to EOA (32.8 vs. 72.4 months, p = 0.038).

## Conclusion

In this cohort, adolescent patients with ERA were started on Methotrexate later and discontinued earlier than other groups. There was also comparatively greater use of Sulfasalazine in ERA patients.

At the time of this study, use of biological agents, especially Infliximab and Adalimumab was significantly higher in ERA. Furthermore, ERA patients were started on biological therapy earlier, once DMARD treatment had commenced; suggesting that escalation of treatment potency was common in addition to switching to alternative biological treatments.

## Disclosure of interest

None declared.

